# GATA2 Regulates Constitutive PD-L1 and PD-L2 Expression in Brain Tumors

**DOI:** 10.1038/s41598-020-65915-z

**Published:** 2020-06-03

**Authors:** Yujie Fu, Connor J. Liu, Dale K. Kobayashi, Tanner M. Johanns, Jay A. Bowman-Kirigin, Maximilian O. Schaettler, Diane D. Mao, Diane Bender, Diane G. Kelley, Ravindra Uppaluri, Wenya Linda Bi, Ian F. Dunn, Yu Tao, Jingqin Luo, Albert H. Kim, Gavin P. Dunn

**Affiliations:** 1 Department of Neurological Surgery, Washington University School of Medicine, St. Louis, Missouri USA; 2 Andrew M. and Jane M. Bursky Center for Human Immunology and Immunotherapy Programs, Washington University School of Medicine, St. Louis, Missouri USA; 3 Division of Oncology, Department of Medicine, Washington University School of Medicine, St. Louis, Missouri USA; 4 Division of Pulmonary and Critical Care Medicine, Department of Medicine, Washington University School of Medicine, St. Louis, Missouri USA; 5 Dana-Farber Cancer Insititute, Boston, Massachusetts USA; 6 Center for Skull Base and Pituitary Surgery, Department of Neurosurgery, Brigham and Women’s Hospital, Harvard Medical School, Boston, Massachusetts USA; 7 Department of Neurosurgery, University of Oklahoma Health Sciences Center, Oklahoma City, OK USA; 8 Division of Public Health Sciences, Department of Surgery, Washington University School of Medicine, St. Louis, Missouri USA; 9 Biostatistics Shared Resource, Siteman Cancer Center, Washington University School of Medicine, St. Louis, Missouri USA

**Keywords:** CNS cancer, Tumour immunology

## Abstract

Encouraging clinical results using immune checkpoint therapies to target the PD-1 axis in a variety of cancer types have paved the way for new immune therapy trials in brain tumor patients. However, the molecular mechanisms that regulate expression of the PD-1 pathway ligands, PD-L1 and PD-L2, remain poorly understood. To address this, we explored the cell-intrinsic mechanisms of constitutive PD-L1 and PD-L2 expression in brain tumors. PD-L1 and PD-L2 expression was assessed by flow cytometry and qRT-PCR in brain tumor cell lines and patient tumor-derived brain tumor-initiating cells (BTICs). Immunologic effects of PD-L2 overexpression were evaluated by IFN-γ ELISPOT. *CD274* and *PDCD1LG2 cis*-regulatory regions were cloned from genomic DNA and assessed in full or by mutating and/or deleting regulatory elements by luciferase assays. Correlations between clinical responses and PD-L1 and PD-L2 expression status were evaluated in TCGA datasets in LGG and GBM patients. We found that a subset of brain tumor cell lines and BTICs expressed high constitutive levels of PD-L1 and PD-L2 and that PD-L2 overexpression inhibited neoantigen specific T cell IFN-γ production. Characterization of novel cis-regulatory regions in *CD274* and *PDCD1LG2* lead us to identify that GATA2 is sufficient to drive PD-L1 and PD-L2 expression and is necessary for PD-L2 expression. Importantly, in TCGA datasets, PD-L2 correlated with worse clinical outcomes in glioma patients.. By perturbing GATA2 biology, targeted therapies may be useful to decrease inhibitory effects of PD-L2 in the microenvironment.

## Introduction

Glioblastoma (GBM) remains a challenging malignancy to treat. Poor clinical outcomes likely reflect aggressive GBM biology^1^, and growing evidence suggests that there is also signficant immunosuppression in GBM. Several immune resistance mechanisms have been described in GBM patients: increased regulatory T cells, indoleamine 2,3 dioxygenase activation, dysregulated antigen presentation, and STAT3-driven myeloid cell suppression, among others^1–3^. Overexpression of inhibitory PD-L1 is another mechanism by which GBM cells may attenuate T cells^4,5^, which is important because PD-1/PD-L1 blocakde is FDA-approved for the treatment of many cancers. In GBM, PD-L1 expression is variable and occurs often without significant infiltrating lymphocytes, suggesting that it may be influenced by tumor intrinsic induction^6^ rather than extrinsic stimulation. Several mechanisms can drive cell-intrinsic PD-L1 induction including *PTEN* loss^7^, aberrant signaling^8^, genomic amplification^9^, and post-translational modifications^10^. However, it is unclear which of these mechanisms is most germane to brain tumors.

The expression of the other known PD-1 ligand, PD-L2, remains underexplored in CNS malignancies and other cancers. Human and mouse PD-L2 were cloned in 2001 and inhibit T cell function^11^. PD-L2 expression was observed in several malignancies^12^ including renal^13^, breast^14^, lung^15^, and gastrointestinal^16^ cancers. Moreover, its expression was associated with worse clinical outcomes in a subset of these cancers^13,14^. However, no studies have documented the expression and clinical relevance of PD-L2 in primary brain tumors.

We characterized PD-L1 and PD-L2 expression in brain tumor cell lines to focus on cell-intrinsic mechanisms regulating their expression. We observed high constitutive expression of PD-L1 and PD-L2 in a subset of brain tumor cell lines and in patient-derived BTICs. We identifed a novel enhancer region active in PD-L1 expression and a novel regulatory region active in PD-L2 expression. Both regions harbored bindings sites for GATA2, whose expression was necessary for PD-L2 upregulation and sufficient for increased expression of PD-L1 and PD-L2. We showed that increased PD-L2 expression correlated with worse clinical outcomes in low and high grade glioma. These data show that PD-L2 is expressed in brain tumors and together with PD-L1 is regulated, at least in part, by GATA2 transcriptional activity.

## Materials and Methods

### Cell culture

The mouse cell line GL261 was obtained from the NCI (Frederick, MD). IOMM-Lee and CH-157 were obtained from Brigham and Women’s Hospital (I.F.D.) and have been sequenced^17^; KNS60, LN464, LN340, YKG1, KALS-1, AM38, and GMS10 were obtained from the Broad Institute (Cambridge, MA). GL261 and human cell lines were cultured in DMEM with 10% FBS. BTICs were generated as described^18^. All cell lines were cultured for fewer than 10 passages. IOMM-Lee, CH-157, LN464, and LN340 were not included in the initial Cancer Cell Line Encyclopedia^19^.

### mRNA expression of PD-L1 and PD-L2 in cell lines

PD-L1 and PD-L2 mRNA expression was examined in the CCLE^19^ by assessing Z-scores in data downloaded from the cBioPortal (www.cbioportal.org)^20,21^.

### Cloning of *CD274* and *PDCD1LG2* regulatory regions

*CD274* and *PDCD1LG2* regulatory regions were inferred by interrogating ENCODE data visualized in the UCSCGB (https://genome.ucsc.edu). Regions were identified based on H3K27Ac peaks, DNase hypersensitivity regions, and CHiP TF data. Constructs were amplified from IOMM-Lee genomic DNA, sequenced, and cloned into pGL3-Promoter. *CD274* constructs: promoter region PD-L1.Pr1 (−4167 to +538), enhancer regions Pr2 (+4564 to +5691) and Pr3 (+8572 to +10276), and Pr3 minimal (+8572 to +9297). *PDCD1LG2* constructs: PD-L2.Pr1 + 2 (−1145 to +780), PD-L2.Pr1 (−1145 to −495), and PD-L2.Pr2 (−525 to +780). Mutated/truncated PD-L2.Pr1 constructs: PD-L2.Pr1(ΔSTAT1) (−1145 to −694) and PD-L2.Pr1(ΔGATA2/3) (−709 to −495). GATA2/3 binding site and STAT1 binding sites were predicted by UCSCGB and PROMO (http://alggen.lsi.upc.es/cgi-bin/promo_v3/promo/promoinit.cgi?dirDB=TF_8.3) TF binding prediction website. PD-L2.Pr1(ΔSTAT1&GATA2/3) (−1145 to −694) was cloned into pGL3 vector.

### mRNA Isolation and qRT-PCR

Total RNA was isolated from cell lines using RNeasy Mini kit (QIAGEN, Hilden, Germany). 1ug of RNA was reverse transcribed using High Capacity cDNA Reverse Transcription kit (Applied Biosystems, Foster City, CA). Quantitative PCR was performed using a CFX96 Real-Time system (Bio-Rad, Hercules, CA) and Taqman gene expression assays for Cd274, Pdcd1lg2, Gata2 and Gata3 (Hs00204257_m1, Hs00228839_m1, Hs00231069_m1, Hs00231119_m1, Hs00231122_m1, respectively Thermo Fisher Scientific). GAPDH was the endogenous control (Hs02786624_g1, Thermo Fisher Scientific).

### Lymphocyte isolation

Lymphocytes were isolated from GL261 tumors as described previously^22^. 1×10^6^ GL261 cells were injected into the flank subcutaneously in 6–10 week old naïve syngeneic C57BL/6 mice. Tumors were harvested when 10 mm in greatest diameter. Tumors were minced into 1–2 mm chunks, plated in 12-well plates and incubated at 37 °C in culture media [RPMI-1640, 1% L-glutamine (200 mM solution in 0.85% NaCl), 1% penicillin/streptomycin, 1% Na pyruvate (100 mM), 0.5% Na bicarbonate (7.5%), 0.1% β-mercaptoethanol (0.05 M), MEM, 10% FBS] with 25–50 U/ml recombinant human IL-2 for 5 days. TILs were harvested and passed through a 70 micron cell strainer. Lymphocytes were purified using the Dead Cell Removal kit (#130090101 Miltenyi Biotec, CA) and CD8 positive selection (StemCell Technologies, MA).

### ELISPOT assay

TIL ELISPOT assays were performed as described previously^22^. 25,000 GL261, GL261.PD-L1, GL261.PD-L2 or GL261.PD-L1/2 cells were plated in 100 μl serum-free C.T.L media per well (Cellular Technology, Ltd., OH) on pre-coated murine IFN-γ ELISPOT plates (Cellular Technology Ltd., OH). Harvested GL261 TIL were added at 15,000 cells/well in 200 μl with Imp3 peptide (10 μM) (Peptide 2.0 Inc., VA). Concavalin A (1 μg/well) was used as the positive control. Plates were incubated at 37 °C overnight and analyzed by C.T.L. ImmunoSpot kit (Cellular Technology Ltd., OH).

### Viral transduction

Retroviruses were generated by transfecting 293 T cells with pCL-Eco and pBabe.puro, pBabe-PD-L1.puro, and/or pBabe-PD-L2 puro and used to transduce GL261 cells. Transduced cells were selected in 2 μg/ml puromycin for 3 days. AM38 and IOMM-Lee were transduced with GATA2 shRNA (Sigma Mission shRNA TRCN0000019264 and TRCN0000019267) lentivirus as described^23^. LN464 and LN340 were transduced with pBabe-GATA2 (Addgene plasmid#1285) and pBabe-GATA3 (cloned from Addgene plasmid#83814) retrovirus.

### Flow cytometry

Cells were stained for PE-conjugated anti-human PD-L2 (Cat#329606, Biolegend) or APC-conjugated anti-human PD-L1 antibody (Cat#329708, Biolegend) at 1:100 dilution on ice for 30 mins and analyzed by BD FACSCalibur. Isotype controls are mouse IgG2a, κ (Cat#400213, Biolegend) and mouse IgG2b, κ (Cat#400322, Biolegend), respectively. GL261 cells overexpressing PD-L1 and/or PD-L2 were stained for PE-conjugated anti-mouse PD-L2 (Cat#107205, Biolegend) and/or APC-conjugated anti-mouse PD-L1 antibody (Cat#124312, Biolegend) at 1:100 dilution on ice for 30 minutes and sorted using a BD FACSAria III cell sorter. Isotype controls were Rat IgG2a, κ (Cat#400508, Biolegend) and Rat IgG2b, κ (Cat#400612, Biolegend), respectively.

### *In vitro* Luciferase assay

Bioluminescence imaging was performed in the Molecular Imaging Center. 4×10^3^ cells/well were plated into 96 well plates. *CD274* and *PDCD1LG2* reporter luciferase vectors and pRLuc-N3 Renilla control were transfected into cells the next day. 48 hours later, D-luciferin and Coelenterazine were added and luminescence was analyzed by an IVIS 50 (PerkinElmer, Waltham, MA; Living Image 4.3.1).

### Statistical analysis

BTIC experiments were repeated (n = 2) and all the other experiments were repeated (n ≥ 3). All statistical analyses were t-test or one-way ANOVA. GBM and LGG clinical and tumor mRNA (RNA-Seq V2) TCGA datasets were downloaded from www.cbioportal.org^20,21^. PD-L2/PD-L1 high and low expression status was defined as expression values ≥ or <median of PD-L2 and PD-L1, respectively (a commonly used, unbiased method of dichotomizing gene expression). Association between expression status of the two genes was examined by Mantel-Haenzel Chi-square test. Kaplan-Meier method was used to estimate empirical survival probabilities and generate KM curves. Log rank test was used to examine statistical significance of differences in OS and DFS between patient groups defined by PD-L1 or/and PD-L2. Hazard ratio with 95% confidence interval (CI) was reported from univariate Cox model. To determine whether PD-L2 expression status was independently associated with survival, multivariate Cox proportional hazards models were used, adjusting for age, gender and *IDH1* mutation. All tests were 2-sided and P-value ≤0.05 was considered statistically significant. All statistical analyses were performed with SAS (version 9.4; SAS, Cary, NC).

### Ethical approval

All procedures performed were in accordance with the ethical standards of the institutional and/or national research committee and with the 1964 Helsinki declaration and its later amendments or comparable ethical standards. Animal studies were approved by the Animal Studies Committee at Washington University. BTIC lines as described^18^ were generated from patients under informed consent in full adherence to tissue banking protocols approved by the Institutional Review Board at Washington University.

### Novelty and impact

We identified high constitutive PD-L1 and PD-L2 expression in brain tumor cell lines and primary BTICs and characterized novel *cis*-regulatory regions for both genes. GATA2 upregulated both ligands and was necessary for PD-L2 expression. High PD-L1 and PD-L2 expression correlated with worse outcomes in GBM and low-grade glioma, while high PD-L2 independently correlated with shorter disease-free survival in low-grade glioma.

## Results

### High constitutive PD-L1 and PD-L2 expression in brain tumor cell lines

**Figure 1:**
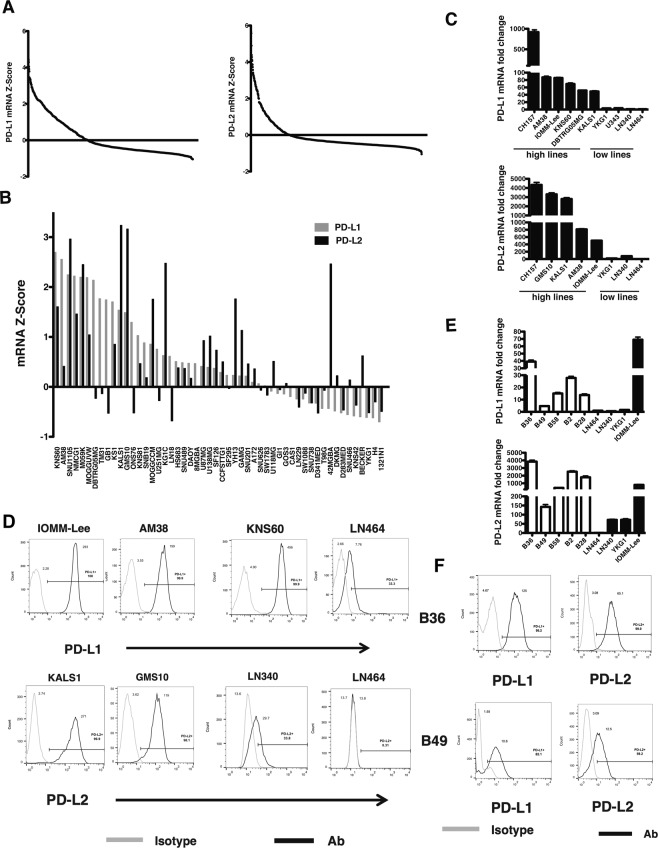
High Constitutive PD-L1 and PD-L2 Expression in Brain Tumor Cell Lines. (**A**) PD-L1 (left) and PD-L2 (right) mRNA Z-scores in the CCLE. (**B**) PD-L1 (grey) and PD-L2 (black) mRNA Z-score in CCLE brain tumor cell lines. (**C**) qRT-PCR validation of PD-L1 (top) and PD-L2 (bottom) mRNA. (**D**) Flow cytometry of PD-L1 (top) and PD-L2 (bottom) expression in brain cell tumor lines (grey line: isotype control; black line: antibody). (**E**) PD-L1 (top) and PD-L2 (bottom) mRNA expression in BTICs by qRT-PCR (white: BTICs; black: brain tumor cells). (**F**) BTIC cell surface PD-L1 and PD-L2 expression (grey line: isotype control; black line: antibody).

We identified cancer cell lines with high constitutive expression of PD-1 ligands to study cell intrinsic, rather than cytokine induced, regulation. We interrogated the Cancer Cell Line Encyclopedia (CCLE), an extensively-characterized human cancer cell line panel^19^. Analyzing 967 cell lines, we identified cancer lines that expressed PD-L1 and PD-L2; 350 cell lines expressed PD-L1 mRNA above the mean expression level, and 193 cell lines expressed PD-L1 mRNA more than 1 standard deviation above all CCLE lines (Fig. 1A). For PD-L2, 220 cell lines expressed PD-L2 mRNA above the mean, and 97 cell lines harbored PD-L2 expression greater than 1 standard deviation above the mean. Cell lines expressing high levels of PD-L1 and/or PD-L2 were derived from all cancers (Supplementary Fig. 1A). Of 52 glioma cell lines, 34 harbored PD-L1 mRNA expression above the CCLE mean whereas 22 cell lines expressed PD-L2 mRNA above CCLE mean levels (Fig. 1B). PD-L1 and PD-L2 expression levels were concordant in some cell lines but divergent in others. To confirm CCLE PD-L1 and PD-L2 expression, we assessed gene expression levels by qRT-PCR in select cell lines with either high or low gene expression. We confirmed high PD-L1 mRNA expression in 6 cell lines relative to 4 low-expressing cell lines (Fig. 1C, top). We also confirmed high constitutive PD-L2 mRNA expression in cell lines including GMS10, KALS1, and AM38 relative to low-expressing LN340 and LN464 (Fig. 1C, bottom). CH-157 and IOMM-Lee meningioma cell lines exhibited high expression of PD-L1 and PD-L2, respectively. Both PD-L1 and PD-L2 cell surface expression levels were evaluated by flow cytometry and were concordant with gene expression (Fig. 1D).

We next examined PD-L1 and PD-L2 gene expression by qRT-PCR in a panel of low passage (≤5 passages from initial culture) patient-derived brain tumor initiating cell (BTIC) lines established from patients with newly diagnosed GBM^18^. In 5 *TERT* mutant BTIC lines^24^, 4 exhibited constitutive PD-L1 and PD-L2 gene expression similar to high-expressing well-passaged cells (Fig. 1E). High PD-L1 and PD-L2 mRNA in the representative B36 BTIC line was concordant with cell surface expression compared to the lower-expressing B49 cell line (Fig. 1F). These data show that a subset of cancer cell lines, including primary BTICs, exhibit high constitutive levels of both PD-L1 and PD-L2. In addition, analysis of TCGA datasets revealed that PD-L2 was also expressed in human cancers including GBM and low-grade glioma (LGG)(Supplementary Fig. 1B), lung adenocarcinoma, melanoma, and renal cell carcinoma (Supplementary Fig. 1C).

### PD-L2 inhibits Neoantigen-Specific T Cell function

**Figure 2:**
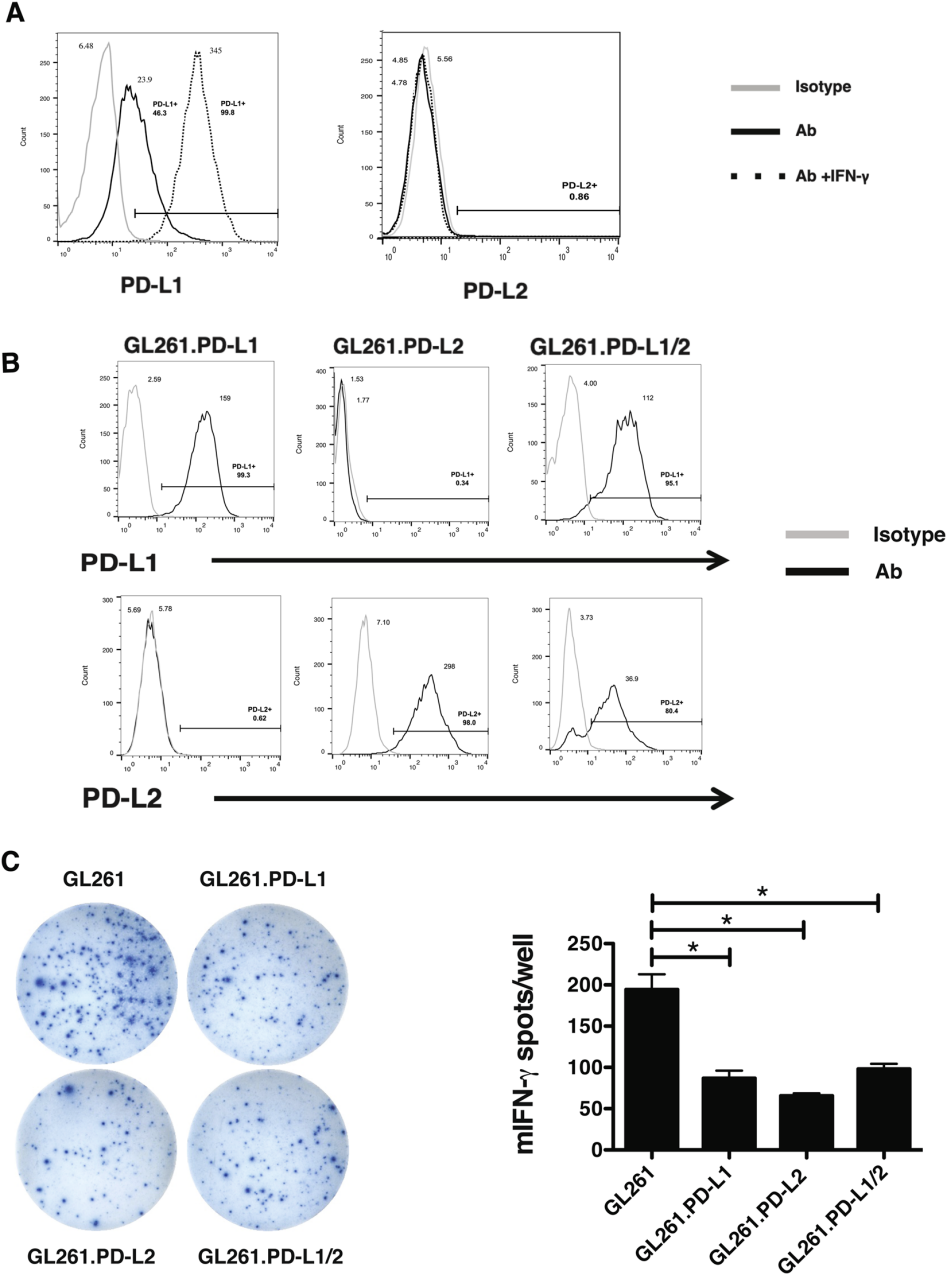
PD-L2 Inhibits Neoantigen Specific T Cell Function. (**A**) Endogenous and IFN-γ inducible PD-L1 (left) and PD-L2 (right) in GL-261 (grey line: isotype control; black line: antibody; dotted line: antibody with IFN-γ stimulation). (**B**) Overexpression of PD-L1 (left), PD-L2 (middle), or both (right) in GL-261 by flow cytometry. (**C**) Representative images from IFN-γ ELISPOT of GL261 TIL with GL261 or GL261 overexpressing PD-L1, PD-L2, or both PD-L1 and PD-L2 (left). Bar graph quantifying IFN-γ spot number per well in ELISPOT (right).

Although the PD-L1 inhibitory functions are well-established, the effects of tumor cell PD-L2 overexpression on T cell function have not been well studied. We tested the effects of PD-L1 and/or PD-L2 overexpression in the GL261 brain tumor model in which the GL261-derived mutant Imp3-D81N 8-mer is an immunogenic neoantigen^22^. GL261 cells express low levels of PD-L1 that increase in response to IFN-γ stimulation, whereas PD-L2 is not expressed or induced (Fig. 2A). To test the effects of both PD-1 ligands on GL261 immunogenicity, we overexpressed PD-L1 (GL261.PD-L1), PD-L2 (GL261.PD-L2), or both ligands (GL261.PD-L1/2) and confirmed their individual and combined overexpression by flow cytometry (Fig. 2B). Compared to parental GL261, each cell line inhibited IFN-γ production by Imp3 neoantigen-specific tumor infiltrating lymphocytes by over 50% (Fig. 2C). Thus, tumor cell overexpression of PD-L2 inhibits neoantigen-specific T cell function similar to PD-L1 overexpression and confirms the inhibitory function ascribed to PD-L2 in non-tumor settings^11^.

### Identification of novel PD-L1 regulatory elements

**Figure 3:**
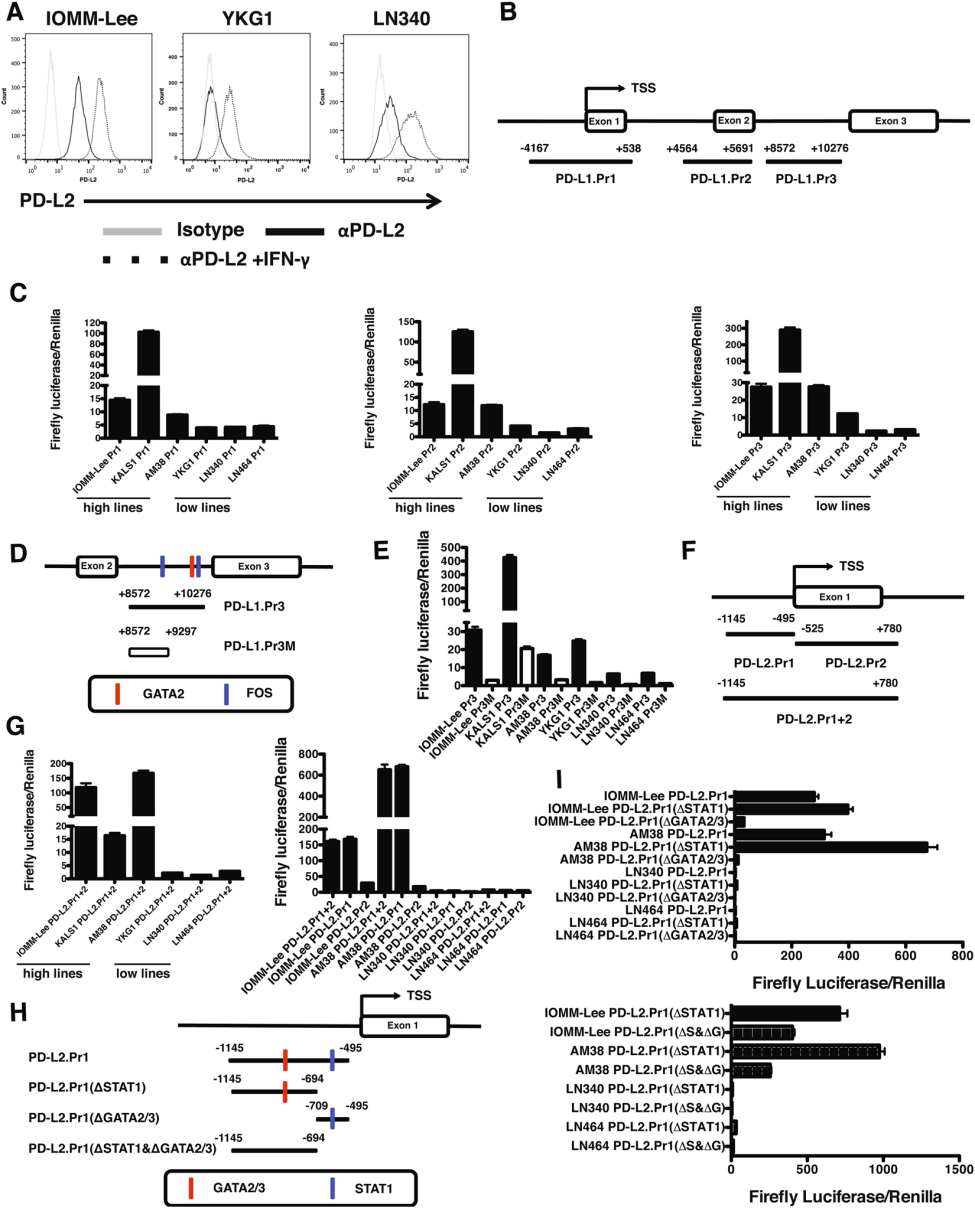
Identification of Novel *CD274* and *PDCD1LG2 cis*-Regulatory Elements. (**A**) Cell surface expression of PD-L2 in brain tumor cells (grey line: isotype control; black line: PD-L2; dotted line: PD-L2 with IFN-γ stimulation). (**B**) *CD274* promoter (Pr1) and enhancer (Pr2 and Pr3) regions cloned and assessed. (**C**) Normalized luciferase activity in cells with high and low endogenous PD-L1 expression using PD-L1.Pr1,.Pr2 and.Pr3 elements. (**D**) Schematic of PD-L1 enhancer region (Pr3) and truncated minimal region (Pr3M). (**E**) Luciferase assay of PD-L1.Pr3 (black) and.Pr3M (white) in PD-L1 high- and low-expressing cell lines. (**F**) *PDCD1LG2* element (.Pr1 + 2) and truncated.Pr1 and.Pr2. (**G**) Luciferase assay of PD-L2.Pr1 + 2 (left) and PD-L2.Pr1 and.Pr2 (right) in PD-L2 high- and low-expressing cells. (H) PD-L2.Pr1 mutants lacking predicted STAT1 (ΔSTAT1), GATA2/3 (ΔGATA2/3), or both binding sites (ΔSTAT1 & ΔGATA2/3). (**I**) Luciferase assay of PD-L2.Pr1 truncations lacking STAT1 (ΔSTAT1) or GATA2/3 (ΔGATA2/3)(top) or also both (ΔSTAT1 & ΔGATA2/3)(bottom) in high and low expressing cell lines.

After confirming that human PD-L1 (Supplementary 2A,B) and human PD-L2 were upregulated by IFN-γ (Fig. 3A), we examined mechanisms that controlled baseline, rather than inducible, PD-1 ligand overexpression. We tested the hypothesis that constitutive expression of PD-L1 and PD-L2 in brain tumor cells is caused, at least partially, by transcriptional activity of *cis*-regulatory elements near transcription start sites (TSS) of *CD274* and *PDCD1LG2*. To identify candidate elements, we examined the University of California Santa Cruz Genome Browser (UCSCGB). The UCSCGB displays human genomic datasets including data from the ENCODE Project^25^. We assessed (a) areas of increased histone acetylation marks (H3K27Ac) suggesting active regulatory regions, (b) regions of increased DNAse hypersensitivity accessible to TF binding, and (c) annotated TF binding sites measured by chromatin immunoprecipitation (CHiP) sequencing. When we integrated these datasets for *CD274*, we identified 3 candidate regulatory elements relative to the TSS using the Genome Reference Consortium Human Build 37: PD-L1.Pr1 (−4167 to +538), PD-L1.Pr2 (+4564 to +5691), and PD-L1.Pr3 (+8572 to +10276) (Fig. 3B). To test their regulatory activity, we assessed their ability to drive luciferase expression. Each element drove luciferase activity in PD-L1 high-expressing lines (IOMM-Lee, KALS1 and AM38) between 3 and 100-fold relative to low-expressing lines (Fig. 3C). Because PD-L1.Pr1 and.Pr2 included regions previously shown to drive PD-L1 expression via ISRE/IRF-1^9^ and AP-1^26^ elements, respectively, we focused on the novel Pr3 region located between exons 2 and 3. To identify the Pr3 region driving activity, we generated a truncation retaining a predicted FOS binding site but lacking 979 bp (PD-L1 Pr3M) including predicted GATA2 TF binding sites (Fig. 3D). In contrast to the full PD-L1.Pr3 construct, luciferase activity in cell lines transduced with PD-L1.Pr3M was decreased by over 10-fold (Fig. 3E). These data show that several *cis*-regulatory regions in *CD274* drive transcriptional activity and identify a novel regulatory region.

### Identification of Novel PD-L2 regulatory elements

Compared to PD-L1, little is known regarding PD-L2 regulation. To determine the molecular basis of constitutive PD-L2 expression observed in our cell line panel, we took a similar approach to our study of *CD274* regulation. Integrating ENCODE data in the UCSCGB, we identified 2 adjacent but distinct candidate regulatory regions defined by H3K27Ac mark density, DNase hypersensitivity, and TF binding sites. We cloned 3 elements from IOMM-Lee cell line genomic DNA relative to the transcription start site: PD-L2.Pr1 (−1145 to −495), PD-L2.Pr2 (−525 to +780), and the PD-L2.Pr1 + 2 region encompassing both sites (−1145 to +780)(Fig. 3F). To determine activity, each was cloned into the pGL3-Promoter luciferase vector and transduced into PD-L2 high-expressing (IOMM-Lee, KALS1, and AM38) and low- expressing (YKG1, LN340, and LN464) cell lines and assayed for luciferase activity. The PD-L2.Pr1 + 2 construct drove normalized luciferase activity 15–150x higher in high-expressing lines compared to low- expressing lines, demonstrating that luciferase activity driven by this element was consistent with endogenous PD-L2 expression levels observed (Fig. 3G, left panel). Moreover, the PD-L2.Pr1 construct drove identical levels of luciferase activity in the high PD-L2 IOMM-Lee and AM38 cell lines compared to the low PD-L2 LN340 and LN464 cell lines. However, when the same cell lines were transfected with the PD-L2.Pr2 constructs, luciferase activity was significantly diminished to about 18% of levels driven by the Pr1 + 2 region in IOMM-Lee and about 2.5% levels driven by the Pr1 + 2 region in AM38 cell lines (Fig. 3G, right panel).

To test the hypothesis that regulatory elements harbored within the PD-L2.Pr1 region were necessary for activity in PD-L2 high-expressing cell lines, we generated mutant constructs of the PD-L2.Pr1 element based on predictions from both the UCSCGB and the PROMO TF prediction resource. Based on TF binding sites that were unique to PD-L2.Pr1 and not found on PD-L2.Pr2, we focused on the GATA2/3 and STAT1 TF binding sites. To test their contribution to PD-L2 Pr1 driven luciferase activity, we generated 3 mutant versions of PD-L2 Pr1 in which (a) the STAT1 binding site was truncated [PD-L2.Pr1(ΔSTAT1), −1145 to −694], (b) the region including the GATA2/3 binding site was truncated [PD-L2.Pr1(ΔGATA2/3), −709 to −495], or (c) the GATA2/3 site was deleted and the STAT1 binding site was truncated [PD-L2.Pr1(ΔSTAT1&GATA2/3), −1145 to −694] (Fig. 3H). The pGL3 constructs were introduced into high- expressing (IOMM-Lee, AM-38) and low-expressing (LN340, LN464) cell lines to determine the effect of each alteration on luciferase activity. The PD-L2.Pr1 construct drove robust luciferase activity in high-expressing cell lines that increased when the STAT1 TF binding site was truncated (Fig. 3I, top panel). In contrast, luciferase activity was nearly extinguished when the GATA2/3 binding site was truncated from PD-L2.Pr1. Luciferase activity was decreased significantly when the GATA2/3 TF binding site was deleted from the PD-L2.Pr1(ΔSTAT1) construct (Fig. 3I, bottom panel). These experiments show that the PD-L2.Pr1 region contains elements that drive constitutive transcriptional activity and suggests that GATA2/3 contributes to this functional role.

### GATA2 Upregulates PD-L1 and PD-L2 and is necessary for constitutive PD-L2 expression

**Figure 4:**
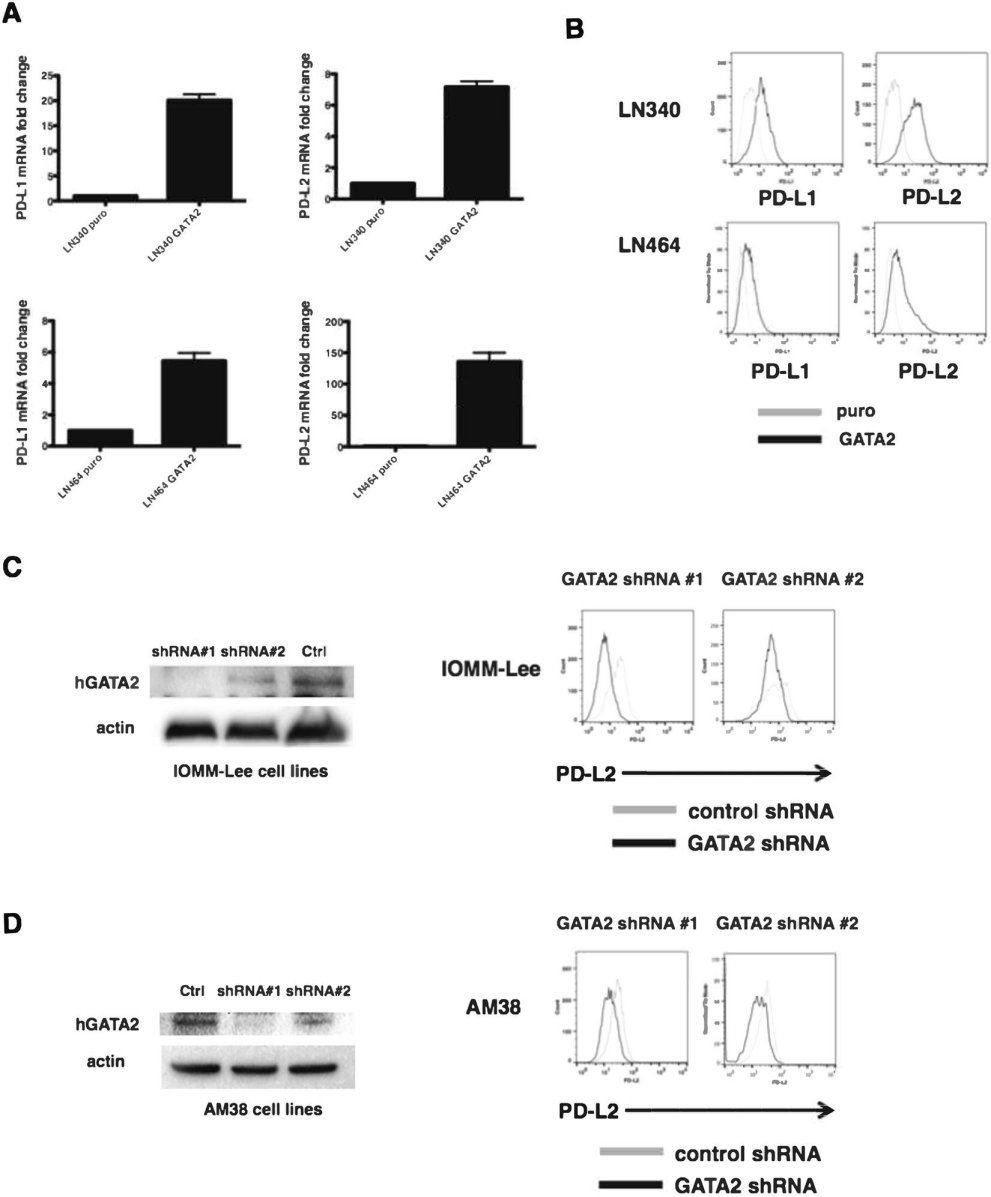
GATA2 Regulates PD-L1 and PD-L2 Expression. (**A**) PD-L1 (left panels) and PD-L2 (right panels) mRNA expression following GATA2 overexpression in LN340 (top panels) and LN464 (bottom panels). (**B**) PD-L1 (left panels) and PD-L2 (right panels) protein expression (grey line: antibody staining in control cells; black line: antibody staining in GATA2-transduced cells) following overexpression of GATA2 in LN340 (top) and LN464 (bottom). (**C**) Knockdown efficiency of GATA2 protein in IOMM-Lee cells by two different individual shRNA contructs measured by western blotting. ShLacZ is used as control vector (left); PD-L2 cell surface protein levels in IOMM-Lee cells transduced with control shRNA (grey line) or shRNA contructs targeting PD-L2 (black line, right panels). The blots were cropped from the same gel and the full blot image is included in the supplementary figure 5A. (**D**) Knockdown efficiency of GATA2 protein in AM38 (left); PD-L2 cell surface protein levels in AM38 cells transduced with control shRNA (grey line) or shRNA contructs targeting PD-L2 (black line, right panels). The blots were cropped from the same gel and the full blot image is included in the supplementary figure 5B.

Because regulatory elements predicted to bind the GATA2/3 TFs were required for high luciferase activity in both PD-L1 and PD-L2 regulatory regions, we tested the hypothesis that GATA2 overexpression was sufficient to drive endogenous PD-L1 and PD-L2 gene and cell surface expression. The LN340 and LN464 cell lines, which expressed low basal levels of these ligands, were transduced with the pBabe.GATA2 or pBabe.puro retroviruses (Supplementary Fig. 3A,B) and assayed for PD-L1 and PD-L2 gene expression and surface protein levels. In both lines, PD-L1 and PD-L2 gene expression measured by qRT-PCR was significantly induced following GATA2 overexpression relative to negative controls (Fig. 4A). Moreover, LN340 and LN464 cells overexpressing GATA2 (Fig. 4B, dark line) upregulated cell surface PD-L1 and PD-L2 by flow cytometry compared to negative controls (Fig. 4B, light line). Because the GATA2 TF binding site shares some overlap with predicted GATA3 TF binding site, we tested the possibility that GATA3 overexpression also drives PD-L2 expression levels. However, when GATA3 was overexpressed, neither PD-L2 mRNA (data not shown) nor cell surface (Supplementary Fig. 3D) changed in LN340 cells compared to the negative control. Thus, overexpression of the GATA2 TF was sufficient to increase PD-L1 and PD-L2 gene expression and protein levels. We next examined whether GATA2 expression was necessary to regulate PD-L1 and PD-L2 levels. To determine the effects of decreased GATA2 expression, we targeted GATA2 transcripts with 2 shRNAs and a control shRNA in IOMM-Lee and AM38 cells (Fig. 4C,D). Although GATA2 overexpression was sufficient to upregulate PD-L1, GATA2 knockdown was not sufficient to decrease cell surface expression of PD-L1 (data not shown). However, GATA2 knockdown using 2 distinct shRNA constructs led to decreased gene expression (Supplementary Fig. 3E) and cell surface expression of PD-L2 in both IOMM-Lee (Fig. 4C) and AM38 (Fig. 4D), PD-L2 high-expressing cell lines concordant with the level of GATA2 protein knockdown observed by western blot. Thus, increased GATA2 expression is sufficient to increase the cell surface levels of both PD-L1 and PD-L2 and is necessary for constitutive high expression of PD-L2.

### PD-1 Ligand expression is associated with worse clinical outcomes in glioma

**Figure 5:**
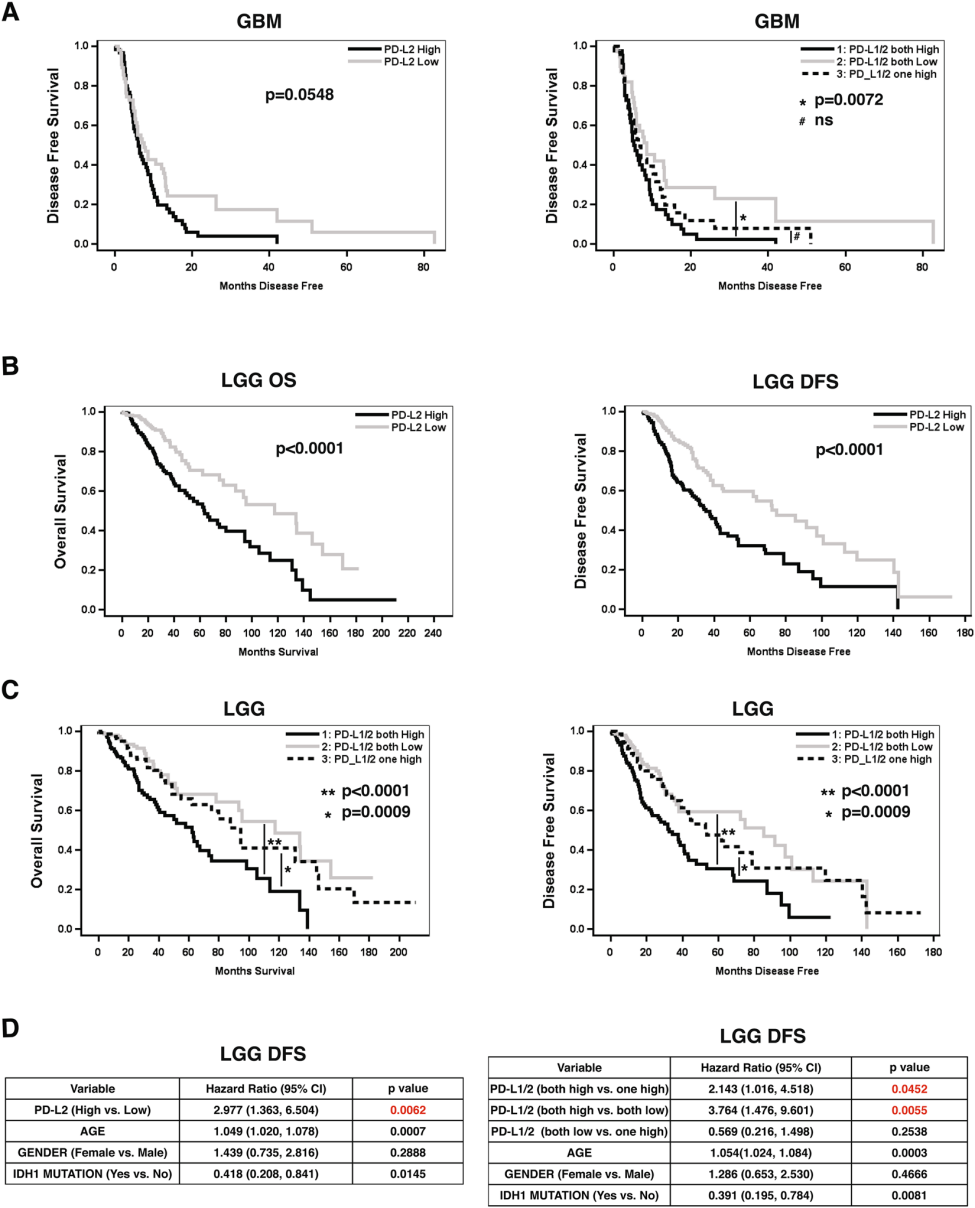
PD-L1 and PD-L2 Expression Levels and Clinical Outcomes in Glioma. (**A**) Kaplan-Meier disease free survival estimates for GBM patients with high expression of PD-L2 (left) and either high expression of both PD-L1/L2, high expression of either PD-L1/L2, or low expression of both (right)(*p = 0.0072; #, ns = not significant). (**B**) Kaplan-Meier estimates of OS (left panels) and DFS (right panels) based on high (dark) or low (grey) PD-L2 expression. (**C**) Kaplan-Meier estimates of OS (left) and DFS (right) in LGG patients with either high expression of both PD-L1/L2, high expression of either PD-L1/L2, or low expression of both (** =p < 0.0001, * = p = 0.009).(**D**) Multivariate analysis for DFS shows PD-L2 is an independent factor in LGG patients after adjusting by covariates of age, gender and IDH1 mutation (p = 0.0062) (left); Multivariate analysis shows PD-L1/2 both high is independently associated with shorter DFS in LGG patients compared to “both low” (p = 0.0055) or “one high” (p = 0.0452) after adjusting by covariates age, gender and *IDH1* mutation (right).

We evaluated the effects of PD-L1 and PD-L2 gene expression on clinical outcomes in both GBM and LGG, brain tumors for which there are genomic and clinical data from TCGA^27,28^. PD-L1 and PD-L2 “high” or “low” expression status was determined by dichotomizing gene expression defined as ≥ or < the median of each gene’s median expression value, respectively. Because we found that expression levels of PD-L1 and PD-L2 were highly concordant in LGG and GBM (Suppl. Fig. 4A), Mantel-Haenszel Chi-square test P < 0.0001), we incorporated PD-L1 expression levels into our analysis. Although individual PD-L1 ligand expression status did not significantly influence disease-free survival (DFS) in GBM (data not shown), GBM patients with high PD-L2 expression showed a trend of shorter DFS than patients with low expression (Fig. 5A, left)(log rank test P = 0.0548). DFS of GBM patients were then stratified by PD-L1 and/or PD-L2 into 3 groups by expression—i.e., both high, both low, or only one highly expressed. GBM patients with high PD-L1/PD-L2 harbored a statistically significant decreased DFS compared to patients who harbored both low expression levels (both high vs. both low: HR, 95% CI = 1.95, 1.21~3.15, log rank test P = 0.0072). However, after adjusting for age, gender, and *IDH1* status, PD-L1 and PD-L2 expression levels alone were not independently associated with disease-free survival in multivariate Cox proportional hazards models (data not shown).

We subsequently tested the hypothesis that PD-L2 and/or PD-L1/2 levels were correlated with worse clinical outcomes in LGG. Patients with high PD-L2 levels exhibited statistically significant shorter overall survival (OS) and DFS compared to patients with low expression levels of PD-L2 (Fig. 5B). LGG patients with both high PD-L1 and PD-L2 expression levels harbored statistically significant shorter OS and DFS compared to patients with either low expression of one or both ligands (Fig. 5C, left and right panels, respectively). In multivariate analsyis, high PD-L2 levels alone were independently associated with worse DFS even after adjusting for age, gender, and *IDH1* status (HR, 95% CI = 2.98, 1.36~6.50, p = 0.0062, Fig. 5D, left), although PD-L2 status alone was not independently statistically significant for OS in LGG (Supplementary 4B). Finally, high dual expression of PD-L1/PD-L2 compared to dual low (p = 0.0055) or singly high PD-L1 or PD-L2 expression (p = 0.0452) was independently associated with shorter DFS when adjusted for age, gender, and *IDH1* status in a multivariate Cox proportional hazards model (Fig. 5D, right). Thus, high PD-L2 expression was associated with worse clinical outcomes in LGG and was an independent prognostic indicator of worse DFS in LGG both alone and with PD-L1.

## Discussion

In this study, we investigated the molecular basis of cell autonomous expression of immunoregulatory ligands PD-L1 and PD-L2. We demonstrated that a subset of cancer cell lines and patient-derived BTICs expresses constitutive high levels of both proteins. By analyzing the genomic regulatory datasets in the ENCODE compendium and interrogating candidate *cis*-regulatory elements in targeted luciferase assays, we identified novel transcriptionally active regulatory elements in both *CD274* and *PDCD1LG2* and showed that GATA2 directly regulates PD-L1 and PD-L2 expression. We also observed that both high PD-L1 and PD-L2 expression was associated with worse clinical outcomes in GBM and LGG; both high PD-L1/PD-L2 levels were independently associated with shorter DFS in LGG.

We analyzed the CCLE to focus on cell autonomous PD-L1 and PD-L2 expression. The CCLE and others have been leveraged typically to study non-immunologic cancer biology. However, the extensive characterization of the CCLE facilitated our studies of PD-L1 and PD-L2, this approach may be useful to study other immunoregulatory ligands. Our observation that a subset of patient-derived BTICs also expressed constitutive PD-L1/2 suggests that this finding was not an artifact of long-term cell passage. Although PD-L1 has been characterized in GBM in recent work^4,5^, PD-L2 expression in GBM has not been described. Ongoing work is directed at extending our observations to fixed tissue. Moreover, although it has been reported that meningiomas overexpress PD-L1, we demonstrate that 2 meningioma cell lines express constitutive levels of PD-L2. While we focused on brain tumors in this study, a subset of all cancer types harbor PD-L2 overexpression. This observation is consistent with a growing number of studies of PD-L2 in other cancers^12–16,29^. Further work is necessary to understand what PD-L2 expression signifies within the broader context of the tumor microenvironment.

*CD274* and *PDCD1LG2* are located adjacent to each other on chromosome 9p24.1, possibly the result of a gene duplication event. However, these genes appear to be differentially regulated. We confirmed previously identified ISRE/IRF-1 and AP-1 TF binding sites 5’ to exon 1 and exon 2, respectively^9,26^ as these regions were contained within our PD-L1 Pr1 and Pr2 constructs. Thus, we focused on a novel region within intron 1 (PD-L1 Pr3). Although we found the GATA2 TF to be sufficient to upregulate PD-L1, nearby genomic regions are also known to contribute to *PD-L1* regulation. In addition, it has become clear that PD-L1 regulation is highly complex. Further work will be important to clarify how these distinct pathways intersect in the regulation of PD-L1.

Compared to PD-L1, the regulation of PD-L2 has been underexplored. Our data corrobrate recent work that interferons upregulate PD-L2^30^, and IL-4 can also induce the expression of PD-L2 in esophageal cancer^16^. We provide evidence that PD-L2 can also be expressed constitutively. We showed that GATA2 is both necessary and sufficient to modulate this expression. GATA2 is one of 6 members of the GATA pioneer TF family involved in development and differentiation^31^. GATA2 regulates stem cell progenitor development and hematopoietic maintenance^32^. GATA2 is also overexpressed in AML and drives prostate cancer pathogenesis, especially in castration-resistant settings^31^. One hypothesis is that GATA2 overexpression drives PD-L2 expression that could inhibit anti-tumor immunity. There are species-specific differences in PD-L2 regulation, and thus further work is necessary in antigenically defined human systems to further define the immunologic consequences of PD-L2 overexpression. Recent work demonstrated that the repulsive guidance molecule b (RGMb) represents another receptor for PD-L2 separate from PD-1^33^, and thus PD-L2 expression may have non-overlapping downstream immunobiological effects from PD-L1. Ongoing work is directed at understanding the molecular basis of GATA2 overexpression and/or upstream pathway activation as well as the other concomitant biological effects its dysregulation may impose in GBM and LGG.

Our data show that PD-L2 transcriptional overexpression correlates with worse clinical outcomes in GBM and LGG. In particular, these data are correlated with decreased DFS in LGG both independently and together with PD-L1 overexpression. Further work is necessary to validate these data on the protein level similar to PD-L1. Additional study is needed to determine the influence of PD-L2 expression on the natural history of brain tumors and to explore whether it may serve as a biomarker of response to immunotherapies. PD-L2 expression has been previously recognized as a biomarker of host immune responses in melanoma^34^, and PD-L2 expression was also predictor of positive clinical responses to anti-PD-1/Keytruda in the Keynote-12 trial for head and neck cancer patients with recurrent or metastatic disease^12^. Because PD-L2 interacts with other receptors such as RGMb, its inhibition may not be achieved exclusively by blocking antibodies to the PD-1 receptor, and thus, there may be merit to exploring efforts to inhibit PD-L2. Finally, it will be important to consider whether the mechanisms that drive constitutive PD-L1 and/or PD-L2 expression—such as GATA2—can be inhibited in a way that decreases the immunosuppressive microenvironment and creates a more permissive context for immune-based therapies.

## Supplementary information

Supplementary Dataset 1.
