# Supplementary material for: GATA2 Regulates Constitutive PD-L1 and PD-L2 Expression in Brain Tumors
Source: Sci Rep. 2020 Jun 3;10:9027. doi: 10.1038/s41598-020-65915-z (PMC7271235; doi:10.1038/s41598-020-65915-z)
Supplement: Supplementary file 1 — Supplementary Dataset 1. [file 41598_2020_65915_MOESM1_ESM.pdf]

Title: GATA2 Regulates Constitutive PD-L1 and PD-L2 Expression in Brain Tumors

Authors and Affiliations: Yujie Fu[1,2], Connor J. Liu[1,2], Dale K. Kobayashi[1,2], Tanner M. Johanns[2,3], Jay K. Bowman-Kirigin[1], Maximilian O. Schaettler[1], Diane Mao [1], Diane Bender[2], Diane G. Kelley[4], Ravindra Uppaluri[5], Wenya Linda Bi[6], Ian F. Dunn[7], Yu Tao[8,9], Jingqin Luo[8,9], Albert H. Kim[1], Gavin P. Dunn[1,2]

[1]Department of Neurological Surgery, Washington University School of Medicine, St. Louis, Missouri

[2]Andrew M. and Jane M. Bursky Center for Human Immunology and Immunotherapy Programs, Washington University School of Medicine, St. Louis, Missouri

[3]Division of Oncology, Department of Medicine, Washington University School of Medicine, St. Louis, Missouri

[4]Division of Pulmonary and Critical Care Medicine, Department of Medicine, Washington University School of Medicine, St. Louis, Missouri

[5]Dana-Farber Cancer Insititute, Boston, Massachusetts

[6]Center for Skull Base and Pituitary Surgery, Department of Neurosurgery, Brigham and Women's Hospital, Harvard Medical School, Boston, Massachusetts

[7]Department of Neurosurgery, University of Oklahoma Health Sciences Center, Oklahoma City, OK, United States.

[8]Division of Public Health Sciences, Department of Surgery, Washington University School of Medicine, St. Louis, Missouri

[9]Biostatistics Shared Resource, Siteman Cancer Center, Washington University School of Medicine, St. Louis, Missouri

Supplementary Figure 1

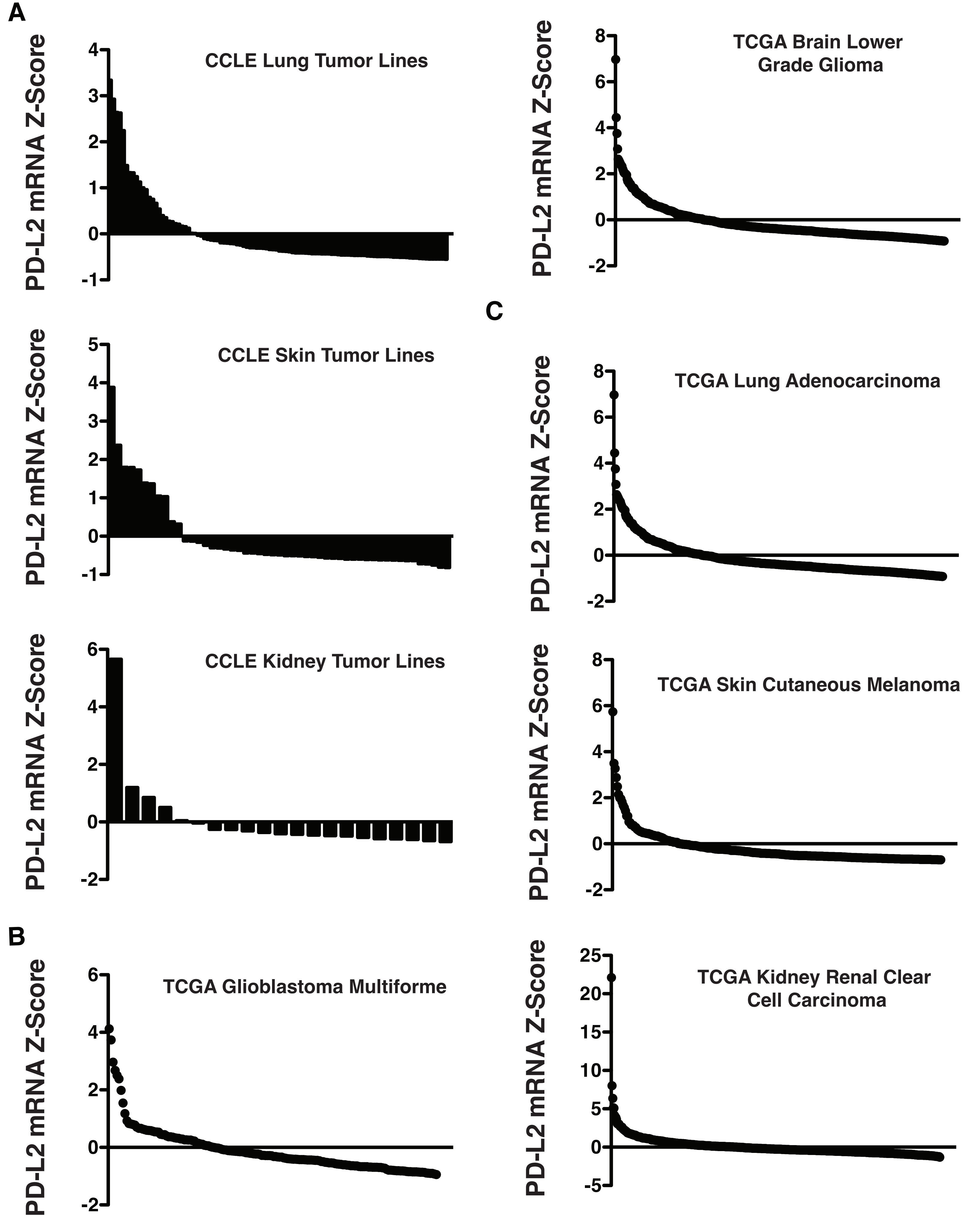

Supplementary Figure 2

A

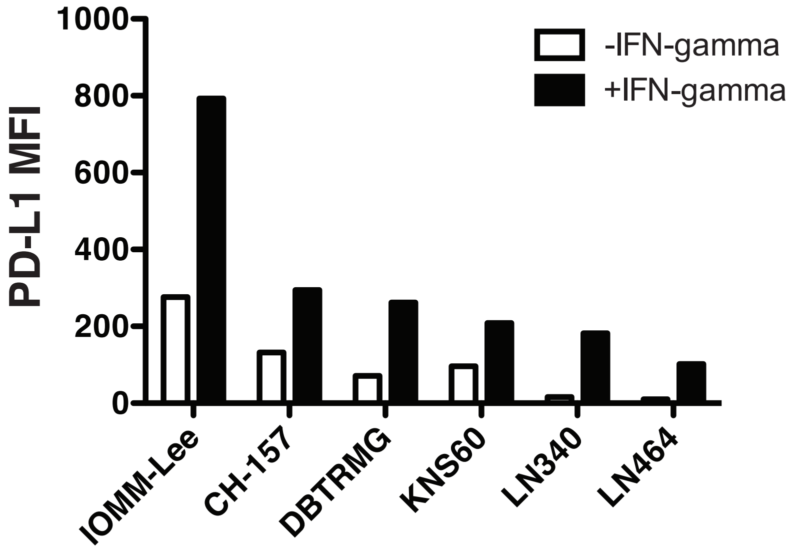

B

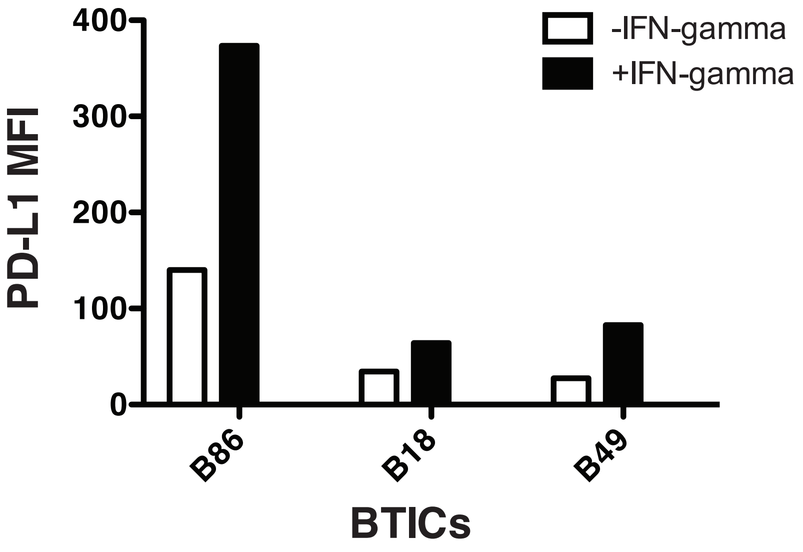

Supplementary Figure 3

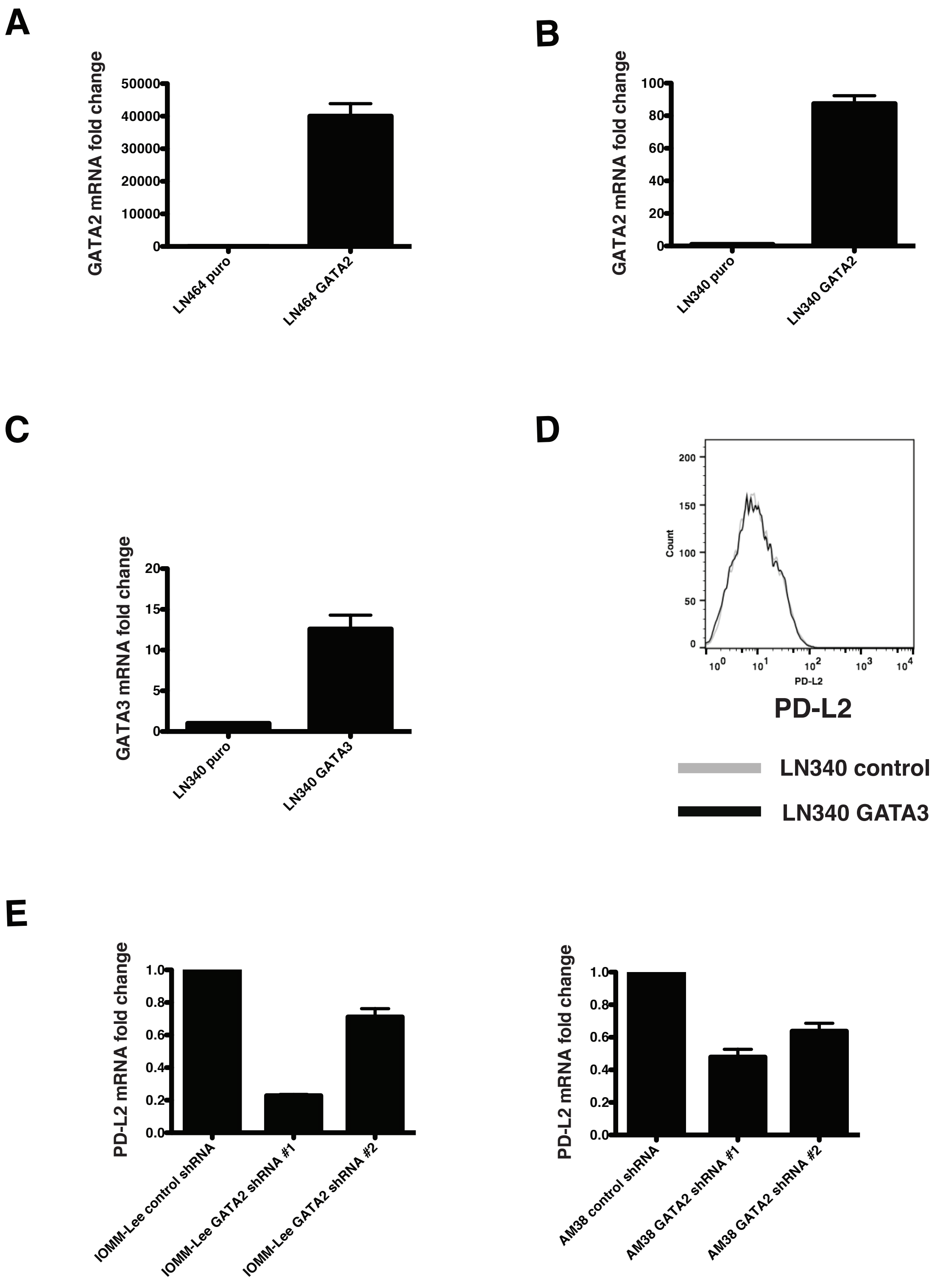

Supplementary Figure 4

A

Table of PD-L2 by PD-L1 expression status (cutoff median of Z scores)			
Frequency Row Pct	PD-L1 Low	PD-L1 High	Total
PD-L2 Low	183<br>69.32%	81<br>30.68%	264<br>50%
PD-L2 High	81<br>30.68%	183<br>69.32%	264<br>50%
Total	264<br>50%	264<br>50%	528<br>100%

LGG N=528, p<0.0001

Table of PD-L2 by PD-L1 expression status (cutoff median of Z scores)			
Frequency Row Pct	PD-L1 Low	PD-L1 High	Total
PD-L2 Low	57<br>69.51%	25<br>30.49%	82
PD-L2 High	25<br>30.12%	58<br>69.88%	83
Total	82	83	165

GBM N=165, p<0.0001

B

LGG OS

Variable	Hazard Ratio (95% CI)	p value
PD-L2 (High vs. Low)	1.746 (0.592, 5.150)	0.3128
AGE	1.045 (1.003, 1.089)	0.0369
GENDER (Female vs. Male)	0.756 (0.287, 1.990)	0.5714
IDH1 MUTATION (Yes vs. No)	0.233 (0.083, 0.652)	0.0056

Supplementary Figure 5

A

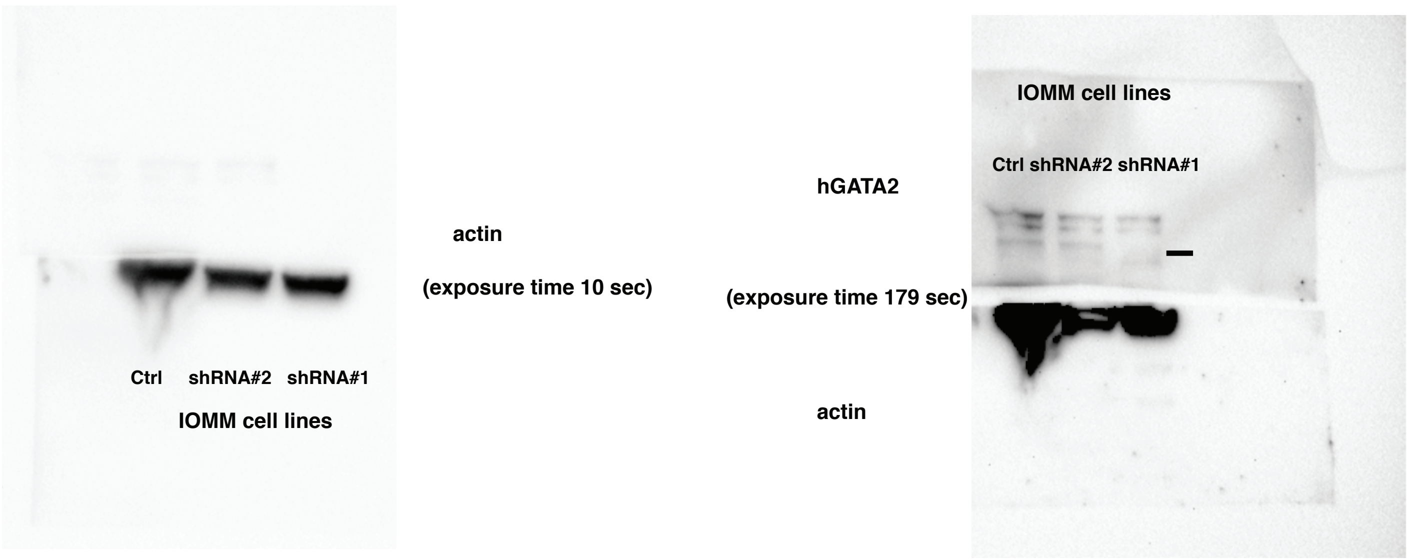

B

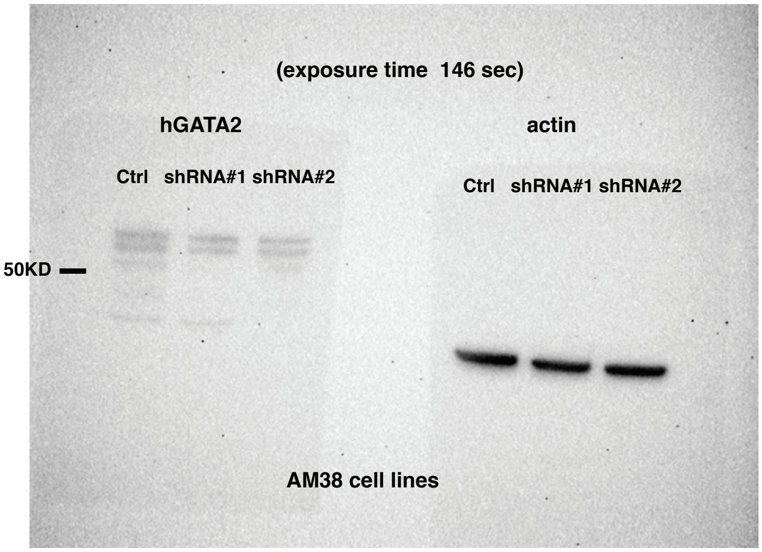

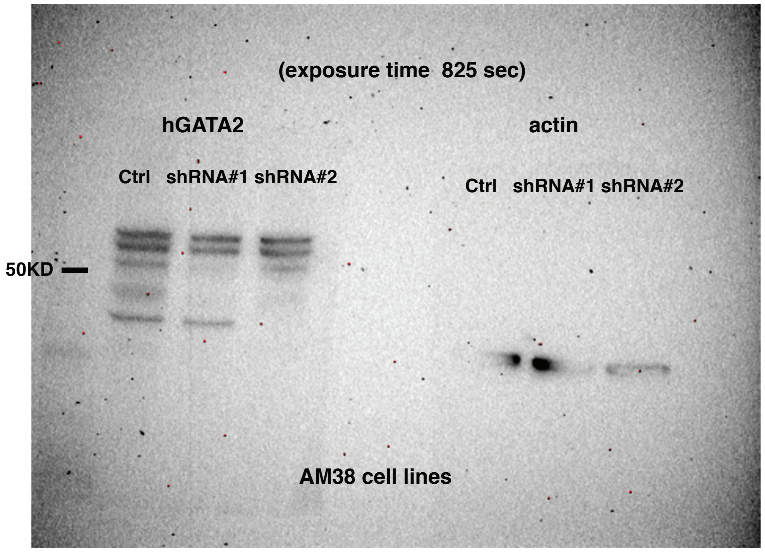

Supplementary Figure Legends

Supplementary Figure 1. PD-L2 expression across cancer types. (A) PD-L2 mRNA expression by Z-score in the subset of lung tumor cell lines (top), skin tumor cell lines (middle) and kidney tumor cell lines (bottom) from the CCLE. (B) PD-L2 mRNA expression by Z-score in TCGA GBM and LGG datasets. (C) PD-L2 expression by mRNA Z-score in TCGA lung adenocarcinoma patients (top), TCGA skin cutaneous melanoma patients (middle) and TCGA kidney renal clear cell carcinoma patients (bottom).

Supplementary Figure 2. **IFN-γ induces the expression of PD-L1 in brain tumors.** (A) IFN-γ stimulation of meningioma (IOMM-Lee and CH-157) and GBM (DBMTRMG, KNS60, LN340, and LN464) cell lines induces the cell surface expression of PD-L1 assessed by flow cytometry. (B) IFN-γ stimulation of BTIC lines (B86, B18, and B49) induces the cell surface expression of PD-L1 assessed by flow cytometry.

Supplementary Figure 3. Perturbation of GATA2 and GATA3 levels in brain tumors. Overexpression levels of GATA2 mRNA in (A) LN464 cells and (B) LN340 cells measured by qRT-PCR. (C) Overexpression of GATA3 mRNA in LN340 cells by qRT-PCR. (D) Overexpression of GATA3 in LN340 is not sufficient to induce PD-L2 protein expression by flow cytometry (grey line: antibody staining in control cells; black line: antibody staining in GATA3-transduced cells). (E) PD-L2 mRNA expression in IOMM (left) or AM38 (right) cells transduced with control shRNA or shRNA constructs targeting PD-L2.

Supplementary Figure 4. Multivariate analysis of OS in LGG. (A) Correlation between PD-L2 and PD-L1 expression values in LGG patients (Pearson Correlation Coefficients, N=528, r=0.57466, p<0.0001) (left) and GBM patients (Pearson Correlation Coefficients, N=165, r=0.51527, p<0.0001) (right). (B) Multivariate analysis for OS shows PD-L2 is not an independent factor in LGG patients after adjusted by covariates including age, gender and *IDH1* mutation (p=0.3128).

Supplementary Figure 5. Knockdown of GATA2 protein levels in brain tumors. Knockdown levels of GATA2 protein in (A) IOMM cells and (B) AM38 cells measured by Western Blot. (A) Actin and GATA2 blot images were cropped from the same gel. Left panel is the actin full image and right panel is GATA2 full blot image in IOMM cell lines transduced with control shRNA or shRNA constructs targeting PD-L2. (B) Actin and GATA2 blot images were cropped from the same gel. Left panel is the GATA-2 full image and right panel is actin full blot image in AM38 cell lines control shRNA or shRNA constructs targeting PD-L2. Top panel and bottom panel are in different exposure time.
